# From memory to prospection: what are the overlapping and the distinct components between remembering and imagining?

**DOI:** 10.3389/fpsyg.2014.00856

**Published:** 2014-08-06

**Authors:** Huimin Zheng, Jiayi Luo, Rongjun Yu

**Affiliations:** School of Psychology and Center for Studies of Psychological Application, South China Normal UniversityGuangzhou, China

**Keywords:** memory, prospection, hippocampus, emotion, self, cognitive control, associative learning, creativity

## Abstract

Reflecting on past events and reflecting on future events are two fundamentally different processes, each traveling in the opposite direction of the other through conceptual time. But what we are able to imagine seems to be constrained by what we have previously experienced, suggesting a close link between memory and prospection. Recent theories suggest that recalling the past lies at the core of imagining and planning for the future. The existence of this link is supported by evidence gathered from neuroimaging, lesion, and developmental studies. Yet it is not clear exactly how the novel episodes people construct in their sense of the future develop out of their historical memories. There must be intermediary processes that utilize memory as a basis on which to generate future oriented thinking. Here, we review studies on goal-directed processing, associative learning, cognitive control, and creativity and link them with research on prospection. We suggest that memory cooperates with additional functions like goal-directed learning to construct and simulate novel events, especially self-referential events. The coupling between memory-related hippocampus and other brain regions may underlie such memory-based prospection. Abnormalities in this constructive process may contribute to mental disorders such as schizophrenia.

“Imagination is everything. It is the preview of life’s coming attractions.”

— Albert Einstein

Humans can not only live in the moment but must also revisit their past experiences and experience the imagined future in advance. During the past century, a variety of modern philosophical, psychological, and contemporary works have served to galvanize interest in the relationship between remembering and imagining. For instance, Khalil Gibran noted, “Yesterday is but today’s memory and tomorrow is today’s dream;” James T. McCay argued, “Tomorrow you promise yourself will be different, yet tomorrow is too often a repetition of today;” while Walt Disney Company underlined, “Here you leave today an enter the world of yesterday, tomorrow, and fantasy.” We are not clairvoyants, but the ability to think about and plan for possible future of the world-prospection-helps us to foresee, set goals, pay close attention to, and represent what is yet to come (Buckner and Carroll, 2007; Gilbert and Wilson, 2007; D’Argembeau and Demblon, 2012). Prospection is crucial for our daily life because it allows individuals to ensure their future interests and prevent future losses in advance. Daily life experiences teach us that our concept of the future closely resembles what we have experienced in the past. If you were asked to picture a day in the life you might lead 20 years from now, you might imagine yourself waking up to the sun rising in the east, eating breakfast prepared by a robot, traveling to work in a high-tech vehicle, and so on. The objects, events, logic, emotions, progress of time, and sense of space making up this imagined world are all similar to the features of the world we have known from lived experience, however, much things may be warped by the forces of imagination; even the aliens we tend to imagine resemble human beings in many ways. It is difficult to conceive of a future that is absolutely different from the past that is stored in one’s memory. It is intuitively obvious that we count on our memories to help us conceptually construct the future. When we envision a life in the future or make plans in anticipation of what is ahead, we consciously or unconsciously use our past experiences or acquired knowledge as a suggestive framework on which to construct new ideas about the future. Hence, remembering the past paves the ground for imagining the future.

Although prospection uses elements of memory to form mental images of possible future scenarios, memory alone does not constitute an imagined future. One key difference between thinking about the past and thinking about the future concerns the subjective concept of time. Memory, however, is not the only contributing factor in prospection. We propose that additional processes must kick into carry out prospection, so as to enable, at least, the organization of present actions, the setting of goals, selection among possible solutions and decisions making. One thing to note is that throughout the review we will employ a variety of conceptual terms referring to the processes enabling prospection, including “imagining the future,” “future simulation,” and “future thinking.” Similarly, we also employ the terms “memory,” “remembering the past,” and “retrospection” in an approximately interchangeable manner.

In this review, we examine evidence for the intimate link between imagining the future and remembering the past, from neuroimaging, lesion, and development research. Further, we discuss how the gap between memory and prospection can be bridged by discussing several lines of research which elucidate how prior experiences shape future stimulation. Specifically, this article discusses four key points of focus: (1) the overlapping processes underlying both memory and prospection; (2) the distinct components guiding memory to shape prospection; (3) how studies on psychiatric disorders provide evidence elucidating the unique functions of these distinct processes; (4) a novel model demonstrating the overlapping and distinct components underlying memory and prospection.

## OVERLAP BETWEEN MEMORY AND PROSPECTION

It has been proposed that imagining the future relies on remembering the past (D’Argembeau and Van der Linden, 2004; Busby and Suddendorf, 2005; Addis et al., 2008; Kwan et al., 2010; Mullally and Maguire, 2013). Primary interest in the neural mechanisms of prospection and memory can be traced to neuropsychological observations with respect to different paradigms. Memory and prospection often collectively draw on several components such as visual modality-leads us to move from one place to another (Rosenbaum et al., 2004; Hassabis and Maguire, 2007); and emotions-help to cope with a diversity of situations (D’Argembeau and Van der Linden, 2004, 2006); as well as self-referential processing-helps us to process information into a complex and coherent content (Szpunar et al., 2007). The specific knowledge and exemplars in memory can be utilized to guide prospection.

### NEUROIMAGING STUDIES

**FIGURE 1:**
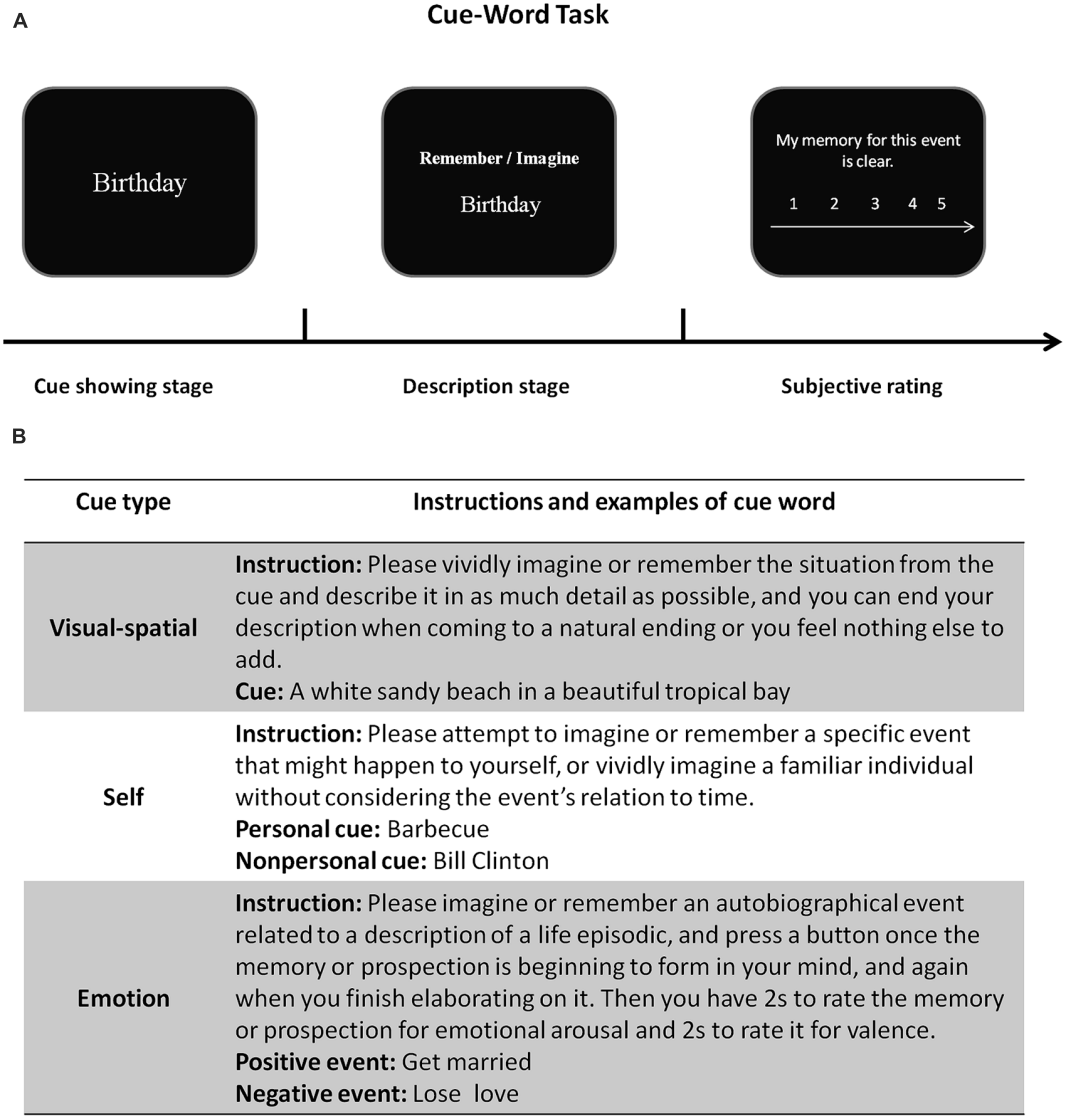
**A typical cue-word task for probing mental conceptions of past and future events. (A)** At the beginning of each trial, an event cue word is presented on a computer screen, whereupon participants are instructed to describe in detail either what they may remember of a specific episode in the past or to imagine a plausible episode in the future. The orienting cue (either to remember or imagine) is shown above the event cue. Following the description stage in which they provide their descriptions as requested, participants rate each episode’s phenomenology (such as its vividness) on a 5-point Likert scale. **(B)** The subsequent panel in **Figure 1** shows primary cue types and corresponding instructions from Hassabis et al. (2007b)), Sharot et al. (2007) and Szpunar et al. (2007). For example, emotional cues contain positive and negative words, and participants are instructed to imagine or remember an emotional event according to the given word and orienting cue.

Recent neuroimaging studies have demonstrated that imagining the future depends on several neural processes involved in remembering the past (D’Argembeau and Van der Linden, 2004; Addis et al., 2007; Buckner and Carroll, 2007; Hassabis and Maguire, 2007; Schacter and Addis, 2007; Schacter et al., 2007; Schacter et al., 2012). There are several psychological processes that are crucial to both memory and prospection and these processes might be associated with the same brain regions in both memory and prospection. Of note, the much used paradigm in experiments is that of cue-word task, as shown below in **Figure 1**.

#### Visual–spatial context

**FIGURE 2:**
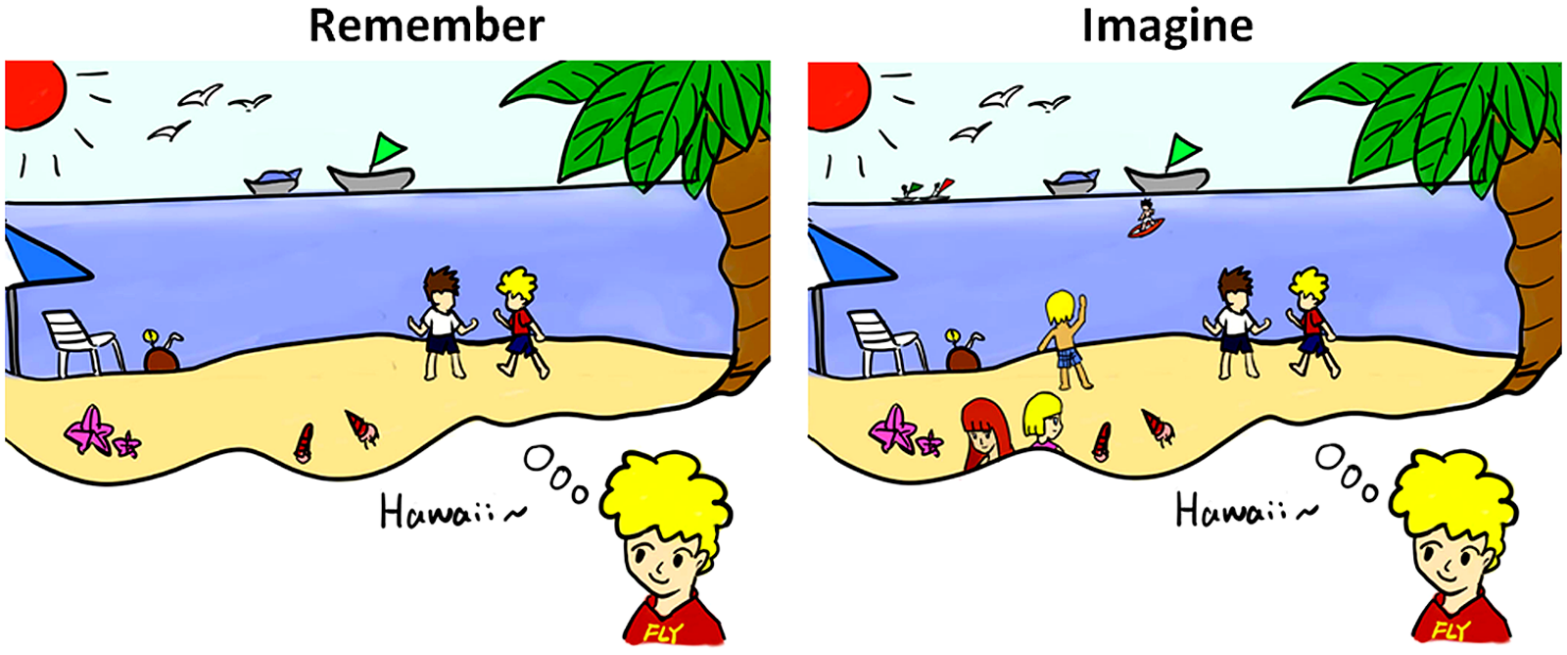
**Examples of memory and prospection concerning visual–spatial context processing.** Once we trace the memory or think about the future, a visual–spatial scene appears in our mind. Constructions of memory and prospection always reflect numerous similarities in scene construction.

A context is widely used as an interpretation of an event; it contains vast information in mind to mediate optimal behavior. The key role of context processing is proactive cognitive control (Barch and Ceaser, 2012; Braver, 2012). It assigns spatial location, temporal information, and further necessary conditions to mediate the memory of the past and the prospection of the future. There are many forms of contexts such as spatial context, temporal context, and cognitive context. Rather essential one is visual–spatial context processing for memory and prospection (Maren et al., 2013). When we remember or imagine a rosy holiday on the beach in Hawaii, everything which has happened or which might happen first comes to our minds: the blissful moment, the captivating sea view, and our lovely companions, as illustrated in **Figure 2**. Such a visual–spatial context provides perceptions and configuration of time and place to frame the memory of the past and on which to construct the scenario of the imagined future.

Memory and prospection share similar visual–spatial contexts including places getting us from one place to another and features of the objects (Rosenbaum et al., 2004; Hassabis and Maguire, 2007). Furthermore, they might both require accessing representations of stored visual stimuli (Rosenbaum et al., 2004). Separate lines of research suggested that visual memory and visual imagery may rely on similar regions (e.g., the frontal-parietal control regions and the occipital–temporal sensory regions; Slotnick et al., 2012). Scene construction is a specific example of visual–spatial associative construction that combines scattered visual elements together to flexibly construct an event as a whole (Hassabis and Maguire, 2007; Maguire and Mullally, 2013). Neuropsychological evidence of lesion with hippocampus confirms that the hippocampus is crucial for memory and navigation (Scoville and Milner, 1957; Spiers et al., 2001). Besides, in contrast to control group, patients with hippocampus amnesia could not imagine new experiences in response to verbal cues outlining a range of common place scenarios (Hassabis et al., 2007b). Scene, in nature, is a highly utilized means of collecting information. In this way, scene construction facilitates the construction of atemporal scenes and forms a foundation with the details of past and future. Hassabis and Maguire (2007) emphasized that scene construction needs visual–spatial context processing, in which memory and prospection both retrieve and provide relevant information into a complex and coherent spatial content.

#### Self-processing

The self is the glue that binds together the past and the future in a consistent manner. To project oneself forward and backward in time is defined as a capacity for “mental time travel” (Suddendorf and Corballis, 2007). With regard to memory and prospection, the self-referential processing covers at least two dimensions: (1) that of representing oneself as an unique individual (e.g., constructing personal scenarios; Szpunar et al., 2007; Abraham et al., 2008) and (2) that of processing scenarios related to personal goals and self-schema (e.g., attaching personal significance to constructed scenarios; D’Argembeau et al., 2010).

The cue-word task, much used in the study of autobiographical memory, is typical of investigations into memory and prospection. In this task, participants get cues (e.g., the cue word: Barbecue) and are instructed to imagine a personal experience of a future event, remember a personally experienced past event, or imagine a specific celebrity (e.g., the cue word: Bill Clinton) with no explicit temporal reference. Specifically, imagining a familiar individual involved neither the self-processing nor the mental time travel, and therefore served as the baseline condition. Szpunar et al. (2007) instructed participants undergoing fMRI to imagine themselves in a plausible future [Self-Future (SF)], remember themselves in a past episode [Self-Remember (SR)], or to imagine a specific event concerning a celebrity [Clinton-Imagine (CI)]. During scanning, participants were required to think about an image as vividly as possible. At the end of experiment, participants finished post-scan questionnaires and rated the phenomenological characteristics (e.g., vividness, emotional arousal, and emotional intensity) of the mental images they had constructed during the procedure. Post-experiment questionnaires indicated that memory and prospection differed in their phenomenological qualities but that they both included conceptions of self in time. Furthermore, a set of regions (e.g., the posterior cingulate cortext (PCC; the hippocampus) revealed no variations in activation during prospection and remembering (although they did display greater overall activity than during the process of imagining a specific event), and these results were dovetailed with studies implicating activation in the frontopolar and the medial temporal lobe (MTL) region (Okuda et al., 2003). However, imagining a non-personal event might rely more on semantic knowledge but not self-referential processing. Imagining the future related to self was associated with activation in the medial prefrontal cortex (MPFC) and the PCC (D’Argembeau et al., 2010). Previous studies linked the MPFC with self-referential processing, and especially coding and evaluating personal goal (D’Argembeau et al., 2005; Schmitz and Johnson, 2006). Moreover, the PCC is related to previously experienced visual–spatial context (Szpunar et al., 2007; Summerfield et al., 2009). Generally, representation of self is the process of projecting oneself into memory. Prospection tends to incorporate more details and can influence a stronger subjective feeling, which is essential to mental simulations.

#### Emotions

Episodic simulations frequently display influence from the emotional affection of whoever is simulating them. Most everyday mental simulations are emotionally arousing, positively or negatively charged. Constructing positive or negative events mentally creates more details than neutral ones (D’Argembeau and Van der Linden, 2004, 2006). People intend to hold a positive self-concept and remember a rosy future during memory (Szpunar et al., 2012). Similarly, people are more likely to be overconfident and optimistic about the future (Sharot et al., 2007). Emotion signals generate specific simulations when retrieving pieces of information from memory (D’Argembeau and Van der Linden, 2007; Schacter et al., 2012). Thus, emotion plays a pivotal role in memory and prospection.

In a fMRI study of optimism (Sharot et al., 2007), participants came up with positive or negative life events (e.g., “get married” or “lose my love”) that happened in the past or that might happen in the future. After scanning, participants rated their subjective feeling toward their memories and projections and also ranked their prevalence of optimistic qualities in them. A set of regions including the MPFC, the PCC, and the amygdala were activated in mental simulations of past and future emotional events. However, activity in the rostral anterior cortex (rACC) and the amygdala was diminished when imagining negative future events rather than positive ones, and was also diminished in remembering of past events despite the use of controls to account for differences in pre-experiencing. Behavioral results also showed that it was only during prospection that participants became more attached to positive events than to negative ones. The MPFC and the PCC contribute to retrieve autobiographical information. Particularly, when imagining positive future events relative to negative ones, the amygdala and the rACC were specifically activated. The effect of arousal on positive events resulted in more activation in the amygdala (Mickley Steinmetz et al., 2010) and the amygdala always modulates memory and decision making. An important role in the ACC is assessing and regulating emotional and motivational information so that people can pay attention to positive future scenarios (Sharot et al., 2007). In conclusion, for both memory and prospection, representations of positive episodes are closely connected to a more powerful subjective feeling than negative ones; this emotional arousing, to some extent, can help human motivate their future decisions and plan to maximize the probability of achieving certain goals (D’Argembeau and Van der Linden, 2004).

Taken together, these studies suggest that both memory and prospection are dependent on visual–spatial context, self-processing and emotions (Hassabis and Maguire, 2007; Szpunar et al., 2007; Maguire and Mullally, 2013). Such a close link between memory and prospection suggests that the dysfunction in one system may be correlated with the malfunction in another system.

In addition, we have provided a summary of the reviewed and discussed neuroimaging studies in **Table 1**. **Table 1** includes a summary of task, the overlapping brain regions between memory and prospection, and the distinct brain regions between memory and prospection.

**Table 1:** Summary of memory and prospection in neuroimaging studies.

		Brain region		
Study	Task	Overlap between memory and prospection	Memory > prospection	Prospection > memory
Okuda et al. (2003)^*^	Future and past talking tasks	Frontal and medial temporal lobes	Right medial frontal cortex and left medial frontal cortex	Most areas in MTL and anteromedial FP
Sharot et al. (2007)	Future and past events tasks	Ventral medial PFC, PCC, and bilateral AMG	DMPFC	Positive future: AMG and rostral ACC
Addis et al. (2007)	Event tasks	Left HC and posterior visuopatial regions	Not revealed	Right frontopolar peel, left ventrolateral PFC, and right HC
Hassabis et al. (2007a)	Recall, recreate, and imagine	Bilateral HC, PHG, RSC, PCu, PPC, and ventral medial PFC	Right thalamus, temporal cortices, and MPFC	Ventral medial PFC
Szpunar et al. (2007)	Self-future, self-remember, and Clinton-imagine	Bilateral PCC, bilateral PHG, and left occipital	Not mentioned	Left lateral premotor cortex, left PCu, and right posterior cerebellum
Addis and Schacter (2008)	Past-and future-event tasks	Left MTL, left posterior HC, and left PHG	Right PHG	Left anterior HC, bilateral HC, right AMG, and right PHC
Botzung et al. (2008)	Past and future events evocation	Medial PFC, posterior regions, and MTL	HC and anterior medial PFC	Not mentioned
Addis et al. (2009a)	Autobiographical event tasks	Two subsystems within the core network	HC, PHG, and posterior visual cortex	Medial PFC, inferior FG, and anterior HC
Weiler et al. (2010)	Event imagination and memory	Medial and lateral temporal regions, medial parietal and medial PC, as well as lateral parieto-occipital areas	Right posterior HC, lateral PFC, and PCu	Lateral PFC, PCu, and right posterior HC
Addis et al. (2011a)	Construct past and future events	Not mentioned	Not significant	Viard et al. (2011)Right anterior HC
Viard et al. (2011)	Memory and envision	PCC, PCu, PFC, and HC	HC, occipital and angular gyri	Inferior frontal and lateral temporal gyri
Østby et al. (2012)	Remembering-imagination cue-word task	DMN, local cortical arealization	Right lateral temporal cortex, temporal gyrus	Right hemisphere; left hemisphere

PFC, prefrontal cortex; PCu, precuneus; HC, hippocampus; PHC, parahippocampus cortex; RSC, retrosplenial cortex; PHG, parahippocampal gyrus; PCC, posterior cingulate cortex; PPC, posterior parietal cortex; AMG, amygdala; TPO, temporo-parieto-occipital junction; IC, insular cortex; IFC, inferior frontal cortex; FG, frontal gyrus; MTL, medial temporal lobe; FP, frontal pole; ACC, anterior cingulate cortex; IPL, inferior parietal lobule; DMN, default mode network; TOM, theory of mind. * means PET studies, others mean fMRI studies.

### LESION STUDIES

Recent lesion studies also conclude that memory and prospection share similar processes-patients with amnesia also have impairment in imagining their future (Tulving, 1985; Rosenbaum et al., 2005; Kwan et al., 2010). Numerous studies, especially focusing on amnesic patients, have highlighted the importance of the hippocampus in memory and prospection (Addis et al., 2004, 2009b; Kwan et al., 2010; Maguire et al., 2010; Squire et al., 2010). Hassabis et al. (2007b) asked amnesic patients with hippocampus damage to vividly imagine something fictitious that may happen in the future (e.g., “Imagine you’re lying on a white sandy beach in a beautiful tropical bay”) and then to describe it in details. Results demonstrated that patients with hippocampus damage lacked spatial coherence in scene construction compared with control participants. For example, when participants were asked to generate novel events occurring in the context of an exotic beach, some of them were only able to imagine the sky but the control group could imagine something highly detailed, even integrated scenarios, suggesting that the hippocampus may contribute to imagining new experiences and re-experiencing episodic memory by providing the spatial context for the fragmented elements of an experience (Hassabis et al., 2007a). Other case studies also described a case of H.C. with developmental hippocampus loss whose deficits in recollection and imagination were reflected in the inability to generate past and future events (Kwan et al., 2010). What an interesting observation is that her future description lacked self-relevant information, suggesting that H.C. fail to contextualize imagination with personal information. Similarity, another autobiographical amnesic patient named K.C., who suffered from hippocampus lesions due to a head injury, was also unable to imagine specific episodes in his personal future (Tulving, 1985; Rosenbaum et al., 2005). Hippocampus not only affects spatial and self processing, but also our evaluation of future outcome. White matter volume in the hippocampus is positively correlated with delay discounting severity, indicating that discounting of future events might depend on one’s ability to remember past events or discounting of past rewards (Yu, 2012).

The aforementioned findings seem to conclusively indicate that the hippocampus is involved in retrieving autobiographical memories, imagining fictitious episodes and simulating the future personal events. However, in contrast to the studies mentioned above, several researchers have argued that patients with hippocampus damage still retain future-oriented thinking ability. For instance, research found that P01 did well in tasks of memory and prospection, despite his dense amnesia and hippocampus damage (Aggleton et al., 2005; Hassabis et al., 2007a). Consistent with the case of P01, another patient Jon, with 50% volume loss in his hippocampus, was also able to retain some memory experience and construct future scenarios (Maguire et al., 2010). Specifically, Cooper et al. (2011) used a naturalistic novel autobiographical memory task to assess the ability of past retrieval and imagination of school-age children who had hippocampus damage and autobiographical memory deficits. Results showed that hippocampus volume in patients correlated positively with memory recall task, but there was no significant relation between the hippocampus volumes and scene construction scores. One possibility is that some residual remaining hippocampus is sufficient for patients to construct fictitious and future scenarios (Maguire et al., 2010). Another possibility is that patients suffer from early damage could develop further strategies and that their relatively semantic memory in residual hippocampus may facilitate the future-oriented thinking (Cooper et al., 2011). Over all, it remains unclear why a parallel effect was uncovered in some cases but differential in others.

### DEVELOPMENT STUDIES

In addition to neuroimaging studies and lesion studies, converging lines of evidence from development psychology suggest that memory shares the same processes with future thinking (Suddendorf and Busby, 2003; Busby and Suddendorf, 2005; Addis et al., 2008). Busby and Suddendorf (2005) asked 3-, 4-, and 5-year-old children to report what they did yesterday and what they would do tomorrow. Moreover, children were required to recall events that had not occurred yesterday and would unlikely occur tomorrow. Results demonstrated that children aged 4 and 5 were capable of answering the question while only a minority 3-year-old children could successfully complete the task, suggesting that the development of memory facilitates the development of prospection to some extent. Verbal task, such as simply asking children what they did and what they will do may test children’s language ability, rather than their memory and prospection abilities (Atance and Meltzoff, 2005; Atance and Sommerville, 2014). Therefore Atance and Meltzoff (2005) used stories and pictorial scenes to evoke particular physiological states (e.g., hungry, thirsty, cold). They instructed children to imagine themselves in these scenarios and to choose one item to bring with them into the situation as well as to provide an explanation. When items were semantically associated with the scenarios, the performance of the 3-and 4-year-olds was negatively affected and did not address the future state, whereas the 5-year-olds were outperformed during the process.

Along a similar line, research about older adults also confirms that memory and prospection develop in parallel-with age increase, deficits occur both in the processes of retrieving and imagining (Addis et al., 2008; Gaesser et al., 2011; Rendell et al., 2012). Recent studies used different paradigms like cue-word task (Addis et al., 2008), experimental recombination paradigm (Addis et al., 2010) and autobiographical task as well as semantic-visuospatial control task (Addis et al., 2011b) to assess memory and prospection of older adults. Results indicated that older adults generate fewer episodic details and internal details when recalling and imagining, suggesting age-related simulation deficit to conditions of retrospection and prospection (Gaesser et al., 2011).

Though numerous studies argue that retrospection and prospection ability may co-develop and draw on similar cognitive process, some findings were inconsistent with such conclusion (Busby Grant and Suddendorf, 2009; Suddendorf, 2010). For example, Busby Grant and Suddendorf (2009) asked preschool children to place pictures representing different events at appropriate places in order to assess their capability of distinguishing the times of events. Result showed that subjects aged 3 and 4 performed equally well in the memory task, but subjects aged 4 performed better in differentiating daily events from more remote future events within prospection task, suggesting that ability of memory and prospection do not develop in parallel (Busby Grant and Suddendorf, 2009).

Why do memory and prospection performances vary so widely between the ages of three and four in these various studies? One possibility is that children failed to understand terms referring to future in different experiments (Busby and Suddendorf, 2005). Requiring children to differentiate the times of events in both the past and the future (Busby Grant and Suddendorf, 2009) may test children’s comprehension of temporal distance rather than their ability in memory and prospection. As 4-year-old children have a more developed language ability and comprehension capability, they are more likely to perform better in future-oriented tasks. Another possibility is that different paradigms (verbal task, item choice, item placement) require different amounts of cognitive control, which is involved in thinking about our personal future. In item selecting, children failed to inhibit choosing an item associated with the scene (Atance and Meltzoff, 2005; McCormack and Atance, 2011). It is likely that children did not experience the state requiring anticipation (e.g., children were not currently cold), and they may feel difficult to draw upon this state as an explicit reason for selecting the correct item (Atance and O’Neill, 2005).

In summary, development studies with preschool children and elderly adults reveal a similarity between memory and prospection, but the different definitions of future in different paradigms and the varying degrees of difficulty in various tasks have led to inconsistent conclusions (see **Table 2** for a summary of development studies discussed herein, and **Table 2** includes comparison of age, task, and individuals’ performance on memory and prospection).

**Table 2:** Summary of memory and prospection in development studies.

Study	Age	Task	Performance of memory task	Performance of prospection task
Suddendorf and Busby (2005)	3–5	Item choice	Not mentioned	Age 5 > age 3, Age 4 > age 3
Suddendorf et al. (2011)	3–4	Item choice	Age 3 = age 4	Age 4 > age 3
Richmond and Pan (2013)	3–5	Item choice	Not mentioned	Age 5 > age 3, Age 5 > age 4, Age 4 > age 3
Atance and Meltzoff (2005)	3–5	Item choice	Not mentioned	Age 5 > age 3, Age 5 > age 4, Age 3 = age 4
Atance and Sommerville (2014)	3–5	Item choice	Age 5 > age 3, Age 5 > age 4, Age 3 = age 4	Age 5 > age 3, Age 5 > age 4, Age 3 = age 4
Busby Grant and Suddendorf (2009)	3–5	Item placement	Age 5 > age 3, Age 5 > age 4, Age 3 = age 4	Age 5 > age 3, Age 5 > age 4, Age 4 > age 3
McCormack and Atance (2011)	4–5	Reasoning task	Age 4 = age 5	Age 5 > age 4
Busby and Suddendorf (2005)	3–5	Verbal report	Age 5 > age 3, Age 4 > age 3, Age 4 = age 5	Age 5 > age 3, Age 4 > age 3, Age 4 = Age 5
Atance and Jackson (2009)	3–5	Future thinking task	Not mentioned	Age 5 > age 3, Age 5 > age 4, Age 4 > age 4
Scarf et al. (2013)	3–4	Recombination paradigm	Age 3 = age 4	Age 4 > age 3
Gaesser et al. (2011)	Young: 18–35; Old: 65–88	Pictorial cueTask	Older adult < younger adult	Oder adult < younger adult
Rendell et al. (2012)	Young: 18–27; Old: 65–83	Imagination Task	Not mentioned	Older adult < younger adult
Addis et al. (2008)	Young: 25 ± 5; Old: 72 ± 5, (Mean ± SD)	Cue word Task	Older adult < younger adult	Older adult < younger adult
Addis et al. (2010)	Young: 22 ± 4; Old: 75 ± 6 (Mean ± SD)	Experimental recombination paradigm	Older adult < younger adult	Older adult < younger adult
Addis et al. (2011b)	Young: 20 ± 2; Old: 73 ± 5 (Mean ± SD)	Autobiographical and semantic–visuospatial control task	Older adult < younger adult	Older adult < younger adult

*> means the left group performs better than the right group.*

*< means the left group performs worse than the right group.*

*= means both groups perform equal well*

## DISTINCT COMPONENTS BETWEEN MEMORY AND PROSPECTION

**FIGURE 3:**
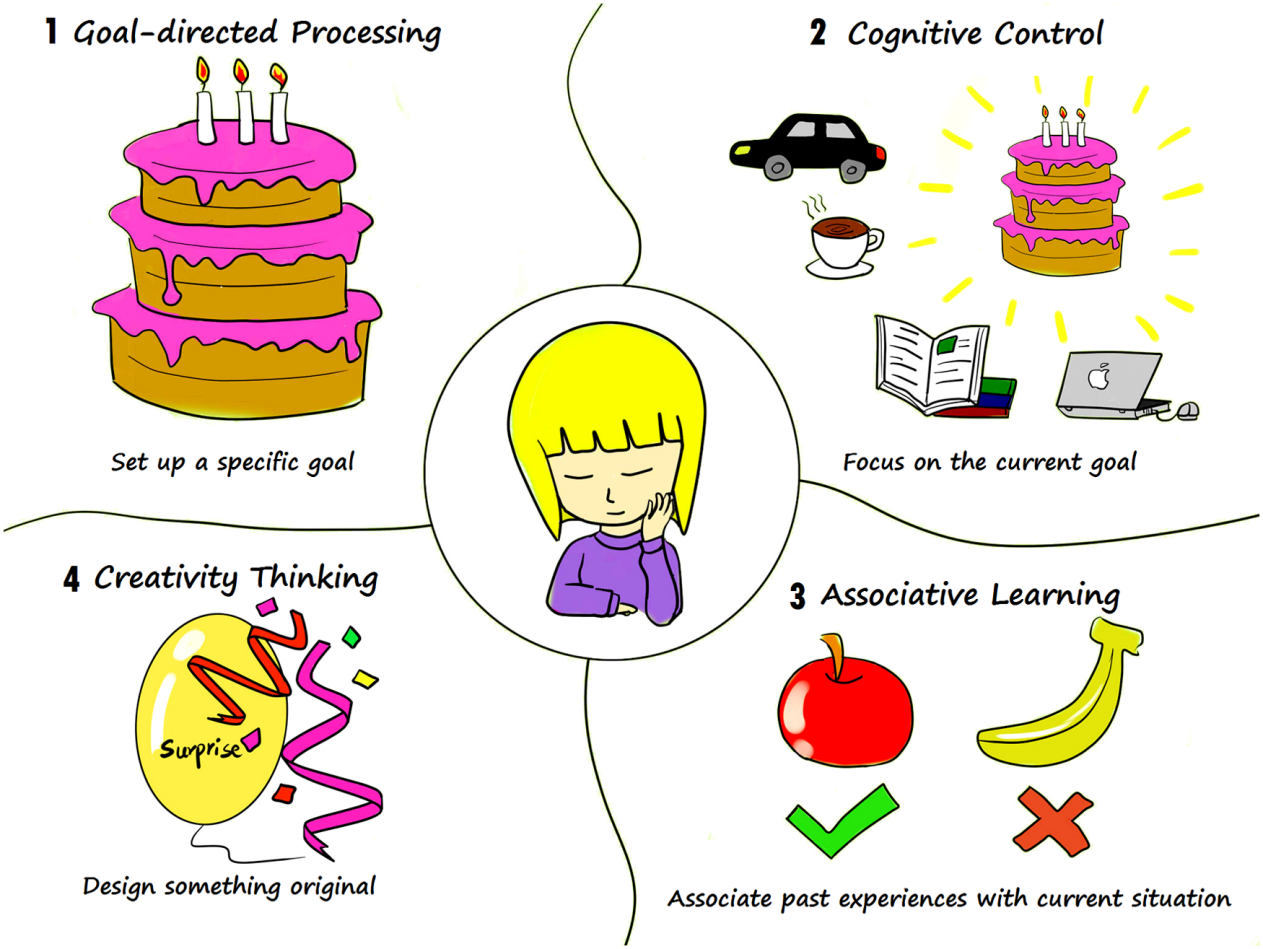
**Distinct processes of prospection.** In order to plan a birthday party, first we should: (1) set a specific goal, such as where to hold the party and who to invite; (2) our planning might next rely heavily on cognitive control to focus on current goal-of holding a birthday party, without regard to other considerations; (3) and eventually in order to generate a more specific plan you might associate with past experiences to better plan your party. An example might go that, having learned that apple pie was more popular than banana pie at your last birthday party, you are now subsequently more likely to prepare apple pie this time around; (4) further to actualize and promote your party, you may also need creativity thinking to facilitate designing something unique.

A forementioned studies have demonstrated the close relationship between memory and prospection. However, memory and prospection are two distinct processes and it is important to tease apart the distinct components of memory and future thinking. To some extent, it has been argued that prospection involves more goal-directed processing, cognitive control, and associative learning as well as creativity (D’Argembeau et al., 2010; Gerlach et al., 2011; Chiu, 2012; Christian et al., 2013). Imagining you are going to hold a birthday party, the first step is to set up a specific goal-when and where to hold a birthday party and who to invite. You might highly in this situation depend on cognitive control to sustain your working memory and to maintain action sequences while planning. For instance, you may have simultaneous, competing wishes to hold a birthday party, take a trip with your family, or enjoy coffee with your friends. But designing a wonderful birthday party keeps you focusing on the current goal and on taking action to promote your project. Then you should make a more definite plan by associating past experiences with your current situation. For instance, if you had learned that apple pie was more popular than banana pie at your last birthday party, you might be more likely to prepare apple pie this time. However, simply representing past experience is not enough; you also need to design something original in order to surprise your guests with your creative thinking (see **Figure 3**).

### GOAL-DIRECTED PROCESSING

Personal goal-setting has been hailed as a shortcut to detail-specific future construction. Prospection is viewed to become goal-directed, with actions like planning, and problem solving (Christian et al., 2013). Besides, goal-directed learning provides more details of problem solutions, even available accesses to information and provides a causal structure to think deeply (Taylor et al., 1998; Gerlach et al., 2011; Christian et al., 2013). However, the neural processing of goal-directed processing and its relation with prospection are little known. Researchers have examined how goal-directed processing shapes prospection and how the concept be discussed across discrete functional domains (Gusnard et al., 2001; D’Argembeau et al., 2010; Niendam et al., 2012).

D’Argembeau et al. (2010) investigated the neural basis for personal goal processing when envisioning the possible events of the future. In this experiment, participants were scanned as they simulated the hypothetical future related to their personal goals (e.g., of getting married next summer) and future events unrelated to their personal goals (e.g., of going to the zoo in 2 weeks). Each of these tasks was compared with a control task only involving the construction of mental representations of intricacies. Results showed that imagining the future related to personal goal elicited stronger activation in the vMPFC and the PCC compared with imagining non-personal future scenarios. The vMPFC and the PCC mediate self-referential processing across evaluation, code and contextualization in mental representation (D’Argembeau et al., 2010). Similarly, a study conducted by Gusnard et al. (2001) concluded that the MPFC had the highest metabolic rate at rest and exhibited decreases across goal-directed behavior. The PCC contributes to prospection contextualization (D’Argembeau et al., 2010), autobiographical information recollection (Svoboda et al., 2006; Spreng et al., 2009) and self-knowledge generation (Northoff et al., 2006). Overall, the vMPFC and the PCC might support the processes that contribute to appraisal, code, and contextualize the future scenarios regarding personal goals and self-schema (D’Argembeau et al., 2010). During autobiographical planning condition, the frontoparietal control network coupled its activity with the default network. Critically, the frontoparietal control network has been associated with executive control processes including planning, initiation, and so forth (Niendam et al., 2012). Therefore, both the default network and the frontoparietal network likely support introspective processes that require goal-directed cognition in self-referential processing.

### COGNITIVE CONTROL

Cognitive control is a special capability of human beings, under which individuals can regulate, coordinate, and sequence their thoughts and actions based on internally maintained behavioral goals (Braver, 2012). According to dual mechanisms of control (DMC) framework, two distinct operating modes modulate the operation of cognitive control: “proactive control” and “reactive control” (Braver et al., 2009). The proactive control mode can be seemed as a form of “early selection,” which helps individuals to maintain goal-relevant information before the occurrence of demanding events and to keep perception, attention and action system in a goal-driven manner (Miller and Cohen, 2001). By contrast, the reactive control is a form of “late correction”, in which attention is only recruited after a high interference event is detected. Therefore, based on interpretation of “proactive control” and “reactive control,” it is easy to link cognitive control with working memory (Baird et al., 2011), problem solving (Monsell, 2003), working memory (Baird et al., 2011), problem solving (Monsell, 2003), as well as planning, and execution (Chan et al., 2008). Take to solve a problem as an example: we need to (1) integrate and sustain more working memory, as well as initiate, organize, and monitor relevant memory to keep the scene and associated problems in mind (Kane and Engle, 2003); (2) envision and encode abstract action sequences which lead to the problem’s solution and maintain movement plans (Gerlach et al., 2011); (3) construct new representations based on the past in order to pre-experience the event (Baird et al., 2011; Kane and McVay, 2013).

Recent research has focused on association between prospection and cognitive control (Kane and Engle, 2003; Okuda et al., 2003). Baird et al. (2011) found that there is a positive correlation between working memory capacity and future-oriented thinking. Working memory is a general cognitive resource, and prospection demands cognitive control, therefore working memory can help individuals to integrate past events stored in memory to construct new representations (Baird et al., 2011; Kane and McVay, 2013). Similarly, evidence from neuropsychological and neuroimaging suggests the default network and the executive network are coactive during prospection (Rowe et al., 2001; Spreng et al., 2010; Gerlach et al., 2011). Gerlach et al. (2011) designed a problem-solving task and instructed participants to solve specific problems in imaginary scenarios. Results revealed activation in the default network and regions associated with executive function and cognitive control, including the DLPFC and the MPFC. Similarity activated regions like the DLPFC are also reported by Spreng et al. (2010), whose study used planning task to test whether the frontoparietal control network would cooperate with the default network to mediate goal-directed cognition. The DLPFC is proved to activate on performance of executive planning task (Rowe et al., 2001), suggesting that cognitive control is in the service of future thinking such as planning and goal-directed processing.

### ASSOCIATIVE LEARNING

The associative theory suggests that learning is driven by a prediction error which is generated by an unexpected outcome or by its unexpected omission (Corlett et al., 2004). The associative processing links retrospection and imagination. It helps individuals to project and imagine a conceivable future scene by integrating past experience in the imaging process. Numerous studies have recognized the close relationship between prediction-error processing and delusion formation (Corlett et al., 2004, 2006; Gradin et al., 2011). Prediction error, the variation between expectancy and actual outcomes, guides adaptive behavior by allocating attention to critical environmental stimuli and creating a casual association between stimuli and environment (Corlett et al., 2006). While patients with schizophrenia, who display deficits in both retrospection and imagination (D’Argembeau et al., 2008) can easily create aberrant beliefs or illusions when a stimulus has not been presented previously but remembered falsely (Johnson, 2006; Corlett et al., 2009). Using associative learning task, Corlett et al. (2007) compared prediction-error-related brain responses in healthy participants and in individuals with schizophrenia. Result showed that the frontal cortex was activated during disrupted prediction-error processing and delusion formation, indicating that schizophrenia inappropriately represents the world with extraneous information. Another study using psychotomimetic drug ketamine found that the greater the magnitude of the DLPFC response is, the more likely individuals experience drug-induced perceptual aberrations or delusion of reference (Johnson, 2006). These lines of evidence indicate that inappropriate engagement of the DLPFC mediates odd perceptions and delusions. The PFC may use information learned in past experience to construct future events via associative learning.

### CREATIVITY

Creativity processing is the sequence of thoughts and actions that leads to innovative generation and adaptive productions (Lubart, 2001). When imagining our future lives, we need creativity thinking to propose novel ideas and to generate unique solution to penitential problem.

Chiu (2012) conducted an experiment using the priming task to assess the association between future thinking and creativity thinking. Participants were required to imagine their life 50 years from now, 5 years from now, and in the present day, respectively. Results showed that creative imagination was better utilized in the 50-year future thinking group. Construal Level Theory suggests that temporal distance changes people’s reaction to future stimuli by reconstructing their mental representations of the future events. Specifically, as the temporal distance increases, events are represented in a more abstract and general way (Liberman and Trope, 1998; Trope and Liberman, 2003). That is to say, in the Chiu (2012) study, individuals considering condition in the distant future engaged in abstract and high-level representations while those prospecting for the near future formed low-level representations. High level and abstract cognitive processing can facilitate creative thinking performance, which implies that even subsequent creative thinking requires abstract thought (Förster et al., 2004).

Comparable levels of activity in MTL regions were observed during both memory retrieval (Squire et al., 2004) and associative processing (Eichenbaum, 2000), which suggests that the MTL network might be important for creativity thinking by associating past experiences with novel idea (Ellamil et al., 2012). The MTL network also underlies both memory and prospection (Schacter et al., 2007; Buckner et al., 2008; Kowatari et al., 2009; Ellamil et al., 2012). It may provide access to stored details, recombine the detail to specific context and encode a simulation to influence and guide future behavior (Addis and Schacter, 2011). The associative function of the MTL network implies that both creativity and prospection share same cognitive process. As future thinking is not just the replication of past memory, but a reconstructed processing, it may cooperate with creativity process to generate unique ideas and to construct novel scenes.

However, we are still concerned that readers may confuse the discreet concepts of prospection and of memory with another relevant concept- “prospective memory.” Prospective memory is a form of memory for an intention to perform a planned action in the future (Okuda et al., 1998; Kvavilashvili et al., 2001) and it is rather prevalent in daily life, including some simple tasks such as remembering to compete in a marathon at 9 am or to take pills to stabilize our condition when we are in the bedroom (McDaniel and Einstein, 2011). Prospective memory and prospection differ on the point that prospection emphasizes foreseeing and planning for the future, while prospective memory concerns remembering to perform intended future events at appropriate time, rather than too much explicit information (Baddeley, 1997; Bayen et al., 2008).

## MEMORY AND PROSPECTION IN PSYCHIATRIC DISORDERS

Complementing the above data, recent neuropsychological studies of psychiatric disorders also provide evidence for distinctions between memory and prospection. Dysfunctions in different processes in remembering and future thinking may result in different psychiatric disorders, on the basis of different causes of deficiency, which can be classified into certain types. For instance, posttraumatic stress disorder (PTSD) is mainly the result of cognitive control and emotional processing dysfunction (Brown et al., 2013a,b); schizophrenia is a result of context processing and self-processing dysfunction (Williams et al., 1996; Klein et al., 2002; D’Argembeau et al., 2008); autism is on account of self-processing impairment (Lind and Bowler, 2010); and Parkinson’s is due to cognitive control deficit (de Vito et al., 2012).

Considerable evidence shows that patients with psychiatric disorders, such as PTSD, autism, schizophrenia, or Parkinson’s, not only exhibit deficits in memory of past experiences, but also are impaired in imagining the future (D’Argembeau et al., 2008; Lind and Bowler, 2010; Brown et al., 2013a,b). PTSD-affected participants tended to recall their memories and imagine future events with less episodic specificity (also be defined as overgeneralization; Brown et al., 2013a,b). Functional avoidance, and executive dysfunction, as well as ruminative thinking might result in patients’ overgeneralized memory (Williams, 2006). Moreover, constructing scenarios during memory may increase perception of future trauma, so individuals with PTSD are more likely to focus on current distress but not past or future, and as a result, generate more overgeneral autobiographical memories than the normal (Brown et al., 2013b).

Patients with schizophrenia, who could not project themselves into specific past and future, possibly due to difficulties in retrieving contextual information from memory, constructing strategic representations, as well as experiencing a continuity of subjective time (Williams et al., 1996; Klein et al., 2002; D’Argembeau et al., 2008). On the whole, schizophrenia may be influenced by impaired self-concept as well as by interference in diverse temporal dimensions of the self (D’Argembeau et al., 2008). Although preliminary, it should be noticed that patients with schizophrenia show greater deficits in prospection tasks than in memory tasks, possibly because of the increasing demand on former processes.

Individuals with autism spectrum disorder (ASD) show attenuation in episodic memory and future thinking, and they are more likely to mentally re-experience past events from an observer (third-person) perspective (Lind and Bowler, 2010). This is probably due to dysfunctions in self-related processing. When patients with Parkinson’s were asked to imagine plausible future episodes, they generated fewer future episodic details than a healthy person (de Vito et al., 2012). On the contrary, patients with Parkinson’s did not show any difference with matched controls in the task of remembering past events, suggesting that the poor performance in the future thinking task is associated with poor executive control but not with impairment of memory (de Vito et al., 2012). Further research should be conducted to explore precise mechanisms of memory and prospection in various patient populations.

## A MEMORY-PROSPECTION MODEL

Remembering and imagining are two radically different processes; however, in our daily life we can utilize memory to construct future scenarios conceptually, which suggests a close overlap between memory and prospection. Past experience or acquired knowledge alone is not sufficient to solve a perceived problem or to create a new idea about the future, indicating that there are several distinct components recruited in prospection. Here, we present a memory-prospection mode to clarify the association and distinction between memory and prospection. In this model, some psychological processes are common to both memory and prospection, including visual–spatial association, self-processing, and emotion. These functions may involve the hippocampus, the MPFC, the amygdala and the insula. The MPFC is linked with self-referential processing and especially with coding and evaluating personal goals (D’Argembeau et al., 2005; Schmitz and Johnson, 2006). The amygdala performs fundamental roles in the storage of memories and decision making associated with emotional events (Sharot et al., 2007).

Beyond its similarities with memory, prospection may also engage goal-directed processing, cognitive control, associative learning, and creativity thinking. Brain regions containing the vMPFC, the dorsal ACC, the PCC, the DLPFC, the precunus, and the hippocampus may be differentially recruited by prospection. The vMPFC and the PCC might support appraisal, code and contextualize the future scenarios with personal goal (D’Argembeau et al., 2010). The DLPFC is related with cognitive control such as planning for the future and achieving a certain goal (Rowe et al., 2001). Besides, the hippocampus, a pivotal part of both memory and prospection, is believed to store information, recombine the information to a specific context, and encode memory to future behavior (Schacter et al., 2007; Buckner et al., 2008).

**FIGURE 4:**
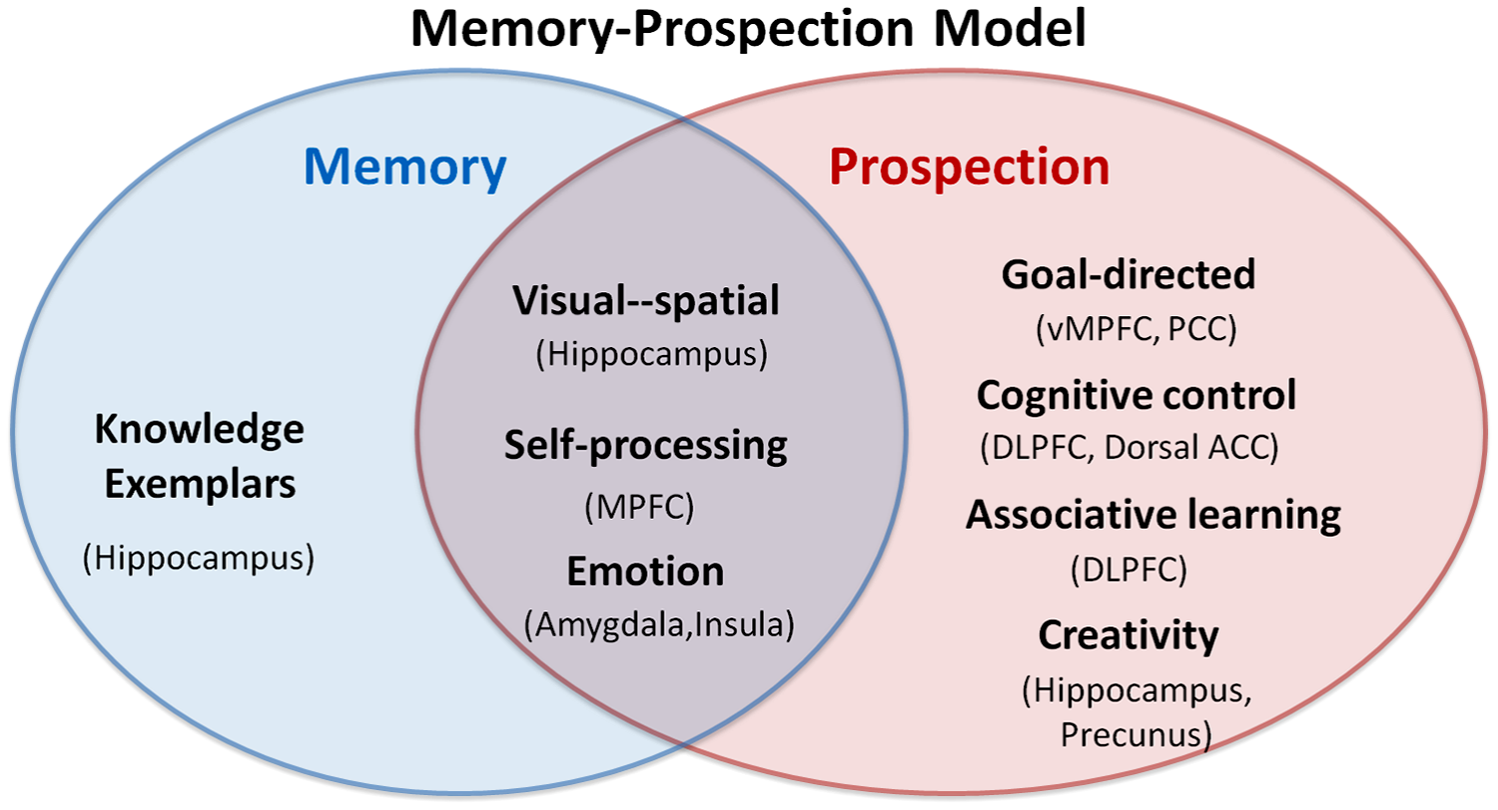
**Interactionist model of memory and prospection.** The overlap and distinct components between memory and prospection are presented in the model; the regions in the parentheses represent corresponding activated regions.

The interactions among these brain regions may contribute to the memory-prospection transformation. We emphasize that these mechanisms are not independent or mutually exclusive but might combine together as complementary ways to envision the past and the future (see **Figure 4**).

## CONCLUSION AND FUTURE DIRECTIONS

In conclusion, evidence from neuroimaging, lesion, development, and psychiatric disorders studies clearly indicates a close relationship between memory and prospection. Both memory and prospection share similar processes including visual–spatial context, self-processing, and emotional activity. Patients with brain lesion and some psychiatric disorder show co-occurring deficits in memory and prospection, while healthy pre-school children and older adults who fail at remembering also fail to project, indicating that prospection adeptly calls on some common psychological processes to generate new future scenarios. The overlaps suggest that prospection adeptly calls on some common psychological processes to generate new future scenarios. However, memory does not solely constitute prospection. We have discussed distinct components such as goal-directed processing, cognitive control, associative learning, and creativity that may link memory to prospection. We propose an interactionist model for memory and prospection to elucidate how memory is transferred into prospection.

Future research will be needed to know what precise mechanisms enable distinct aspects of the mind to transfer memory into prospection, and how they interact with each other. Functional connectivity analysis is needed to further investigate how different regions of the brain talk to each other in the process of prospection. Finally, there is much yet to examine so as to a more precise interactionist model and a broader understanding of the relationship between memory and prospection.

## Conflict of Interest Statement

The authors declare that the research was conducted in the absence of any commercial or financial relationships that could be construed as a potential conflict of interest.
